# Comparative Analysis of Field-Isolate and Monkey-Adapted *Plasmodium vivax* Genomes

**DOI:** 10.1371/journal.pntd.0003566

**Published:** 2015-03-13

**Authors:** Ernest R. Chan, John W. Barnwell, Peter A. Zimmerman, David Serre

**Affiliations:** 1 Genomic Medicine Institute, Cleveland Clinic Lerner Research Institute, Cleveland, Ohio, United States of America; 2 Center for Global Health and Diseases, Case Western Reserve University, Cleveland, Ohio, United States of America; 3 Malaria Branch, Division of Parasitic Diseases and Malaria, Center for Global Health, Centers for Disease Control and Prevention, Atlanta, Georgia, United States of America; Arizona State University, UNITED STATES

## Abstract

Significant insights into the biology of *Plasmodium vivax* have been gained from the ability to successfully adapt human infections to non-human primates. *P*. *vivax* strains grown in monkeys serve as a renewable source of parasites for *in vitro* and *ex vivo* experimental studies and functional assays, or for studying *in vivo* the relapse characteristics, mosquito species compatibilities, drug susceptibility profiles or immune responses towards potential vaccine candidates. Despite the importance of these studies, little is known as to how adaptation to a different host species may influence the genome of *P*. *vivax*. In addition, it is unclear whether these monkey-adapted strains consist of a single clonal population of parasites or if they retain the multiclonal complexity commonly observed in field isolates. Here we compare the genome sequences of seven *P*. *vivax* strains adapted to New World monkeys with those of six human clinical isolates collected directly in the field. We show that the adaptation of *P*. *vivax* parasites to monkey hosts, and their subsequent propagation, did not result in significant modifications of their genome sequence and that these monkey-adapted strains recapitulate the genomic diversity of field isolates. Our analyses also reveal that these strains are not always genetically homogeneous and should be analyzed cautiously. Overall, our study provides a framework to better leverage this important research material and fully utilize this resource for improving our understanding of *P*. *vivax* biology.

## Author Summary

In this study we compare the genome sequences of *Plasmodium vivax* collected directly from patients with those of parasites propagated in laboratory monkeys. We show that the adaptation and continuous propagation of *Plasmodium vivax* in monkeys does not induce systematic changes in the genome and, therefore, that these parasites constitute an unbiased resource for studying this important pathogen. Our analyses also reveal that some monkey-adapted *Plasmodium vivax* strains are not genetically homogenous and retain multiple genetically different parasites present in the original patient infection. Overall, our study confirms the utility of monkey-adapted *Plasmodium vivax* strains for malaria research but also shows that this resource should be analyzed cautiously as different samples of the same strain might provide different biological material.

## Introduction

Today approximately 2.5 billion people are at risk of *Plasmodium vivax* malaria [1]. While transmission of *P*. *falciparum* is slowly decreasing in many countries committed to malaria elimination, vivax malaria displays surprising resilience in a majority of these countries [2]. This difference, likely resulting from the important biological differences between the two parasite species (e.g., the existence of a dormant stage in *P*. *vivax*), calls for specific elimination strategies targeting *P*. *vivax* more efficiently. However, our understanding of *P*. *vivax* biology remains limited by the difficulties of culturing *P*. *vivax in vitro*. The lack of an *in vitro* culture system notably hampers investigations of parasite cell and developmental biology, biochemistry, and the physiology of host cell and parasite interactions by decreasing the availability of the parasite to most laboratories. Rapidly advancing genomics technologies have led to a growing number of *P*. *vivax* whole genome sequences [3–6]. In-depth characterization of multi-gene families [3], identification of single nucleotide polymorphisms [3,5], gene rearrangements [7] and previously uncharacterized genes [8] have for example, provided the molecular foundations to prompt new hypotheses and studies on this important parasite. However, testing these hypotheses *in vivo* remains difficult and, currently, our best opportunity to investigate *P*. *vivax* biology may be through *P*. *vivax* parasites that have been adapted for propagation in New World monkeys [9].

Monkey-adapted *P*. *vivax* strains are typically generated by direct injection of parasitized erythrocytes from patients or, after passage through mosquitoes, by the injection of sporozoites dissected from infected mosquito salivary glands into *Saimiri* or *Aotus* monkeys [10]. Once infections are stably established by serial passage, these strains can be continuously propagated in monkeys by initiating further infections using sporozoites or infected erythrocytes, which can be cryopreserved for later use. These parasites are extremely useful to obtain large amount of proteins or nucleic acids from a single strain and can be shared among researchers to investigate various aspects of the parasite biology. However, important questions regarding their biological relevance and homogeneity remain unanswered.

It notably remains unclear whether the host switch, from humans to New World monkeys, induces or requires specific genomic changes. While *P*. *vivax*-like parasites have been identified in great apes, to date, genomic studies have indicated that these parasites belong to a clearly distinct sister clade, basal to the human *P*. *vivax* [11] and suggest that *P*. *vivax* are specific to humans. In addition, many attempts to adapt *P*. *vivax* to New World monkeys fail to result in detectable levels of the parasite [12], alter the parasite life cycle [13] or are only successful in a specific monkey species or subspecies [13,14]. (Note that once a strain has been successfully adapted, it can typically be more easily propagated in subsequent monkeys.) These observations suggest that the molecular mechanisms used by *P*. *vivax* to invade and survive the metabolic environment of red blood cells (RBCs) and evade the host innate and adaptive immune responses have been tuned to humans by thousands of years of evolution and might be maladapted to New World monkey physiology and RBCs. Successful adaptation to the new environment of New World monkey RBCs could therefore require subtle changes throughout the genome. Interestingly, *P*. *vivax* does not seem to be able to infect Old World monkeys, although these primates are more closely related to apes than the New World monkeys. On the other hand, there are clear indications that Old World primate malaria parasites can infect humans [15–18] despite consequent differences in genome sequences [19,20]. Note however that these infections are not usually as robust as in the natural hosts and that these parasites’ genomes have not been examined after passage in humans. Independently of the host switch, the continuous serial blood stage propagation of adapted parasites in New World monkey may also induce genomic changes as some genes become dispensable in this setting. For example, the Vietnam IV Palo Alto strain is not able to infect mosquitoes [21] suggesting that some genes underlying infectivity to mosquitoes might have been altered during propagation in monkeys. In this regard, it is important to note that many genomic rearrangements have been documented during the propagation of *P*. *falciparum* in *in vitro* cultures [22,23]. Finally, once an isolate becomes a monkey-adapted strain it is often unknown whether it consists of a single homogeneous clonal parasite population (i.e., a single “genotype”, later referred to as a clone) or a complex infection as observed in genomic analysis of field isolates [5] and numerous field studies (see e.g., [24]).

In this study, we compare the genomes of seven monkey-adapted strains with the genomes of six field isolates to characterize genomic changes that potentially occur during adaptation to New World monkeys and continuous propagation. We also analyze six different samples collected during the generation of the Mauritania-I and Mauritania-II strains. These analyses provide additional insights regarding the homogeneity of monkey-adapted strains and the changes that occur during the establishment and propagation of these strains.

## Methods

### Samples

For our analyses, we used genome sequence data previously generated from seven monkey-adapted strains: the Salvador-I [25], Belem [5], Chesson [8], Brazil-I [3], India-VII [3], Mauritania-I [3], and North Korean [3] strains. We compared these sequences with data from six previously sequenced field isolates from Cambodia and Madagascar (M08, M15, M19, C08, C15, and C127) [5,7]. For some of the analyses, we focused on four of these field isolates (M08, M15, C08, and C127) that carry one single highly dominant clone and therefore allow inference of the entire haploid genome sequence (see supplemental information in [5] for details). Several sequencing runs were independently produced for the samples sequenced at the Broad Institute and we used, for most of our analyses, those generated using 101 bp paired-end reads (as these are most similar to the data we generated). The remaining libraries were only used to assess sequencing error hotspots and unannotated paralogous sequences (see below). Detailed information on the samples and sequencing libraries used is provided in S1 Table.

In addition, we analyzed sequences from DNA extracted from additional blood samples collected during the generation of the Mauritania-I and Mauritania-II *P*. *vivax* strains [26]. Three blood samples (AI-3221, AO-521 and WR-1714) were collected from *Aotus nancymaae* monkey infections derived directly from the original patient infection in February 1995. The infection in WR-1714 was initiated by sporozoites collected from mosquitoes fed on blood from the patient’s initial infection. DNA of the stabilate of the Mauritania-I strain sequenced by the Broad Institute [3] came from infections of two *Saimiri boliviensis boliviensis* monkeys, SI-3095 and SI-3097. We also analyzed the blood sample from the patient when a relapse occurred in October 1995 and blood from an *Aotus nancymaae* monkey (AI-3218) infection derived from this relapse after five direct serial passages in monkeys. The AO-521, WR1714, AI-3218 and patient samples were collected in 1995 and cryopreserved at the Division of Parasitic Diseases of the Centers for Disease Control and Prevention (Atlanta, GA). The AI-3321 specimen was collected in 2006 from a monkey infected by parasitized erythrocytes from AI-653 (that had been cryopreserved since 1995). For all samples, we extracted DNA from 200 μl of cryopreserved blood using the Qiagen DNeasy Blood and Tissue kit according to the manufacturer’s instructions.

### Analysis of whole genome sequence data

We mapped sequencing reads from all samples to the *P*. *vivax* Salvador-I [25] reference genome using bowtie2 [27]. We mapped each end of all read pairs independently and considered as correctly mapped only reads best mapped to a single genomic location. Only read pairs for which both ends fulfilled this criterion were included for further analyses. We also identified read pairs that mapped to the exact same positions and randomly discarded all but one pair to eliminate reads representing DNA molecules amplified during the library preparation. In total we examined 13 strains; seven monkey adapted isolates and six human field isolates.

### Identification of Single Nucleotide Variants throughout the *P*. *vivax* genome

We screened for single nucleotide variants (SNVs) at all nucleotide positions covered by at least 20 reads with a base quality score greater than 30 in all analyzed samples. Regions of high DNA sequence similarity were excluded from our analysis as previously described [5]. Overall, 19.7 Mb or 87% of the Salvador-I reference genome sequence were analyzed. Mismatches (i.e., SNVs) between reads generated from a given sample and the reference genome sequence were determined using samtools mpileup [28] and the extended base alignment quality computation. Positions were considered variable only if at least 10% of the reads from a given sample supported an allele different from the reference nucleotide.

### Analyses of DNA sequence insertions, deletions and inversions

We screened each genome for DNA sequence rearrangements as described in [7]. Briefly, we analyzed all read pairs that did not map in the expected configuration (i.e., head-to-head within 1 kb from each other) and might be indicative of deletions (reads mapping head-to-head but distant by more than 1 kb), inversions (reads mapping in a head-to-tail configuration) and tandem duplications (tail-to-tail) (see S1 Fig. of [7] for details). We then identified regions of the genome with more read pairs in unusual configurations than we would expect by chance (as modeled by a Poisson distribution). To avoid including artifacts occurring during library preparation, we focused on rearrangements greater than 1 kb but smaller than 100 kb.

We also searched for large deletions by scanning for chromosomal regions greater than 100 kb where the sequence coverage was less than 50% of the genome average coverage of the sample. To avoid including regions where reads systematically mapped poorly (due to high DNA sequence divergence or high repeat content), we restricted our analyses to loci that displayed low sequence coverage in some, but not all, of the samples.

### Identification of minor clone variants and genotyping validation

To identify positions in the *P*. *vivax* genome prone to systematic sequencing errors, we analyzed sequence data generated by the Broad Institute: for some of the monkey-adapted strains, the sequence data came from several independent sequencing reactions generated from the same library (S1 Table). We examined the reference allele frequency (RAF) for nucleotide positions sequenced by more than 50 reads in each of three independent sequencing runs of the Brazil-I, North Korean, and Mauritania-I strains. We then catalogued genome positions that displayed a RAF between 1–10% and 90–99% in all three runs of these three samples. We focused for this analysis on positions where less than 10% of the reads differed from the main allele (i.e., from all other reads) as this corresponds to the peak of RAF observed in Mauritania-I (see Results). These “consistently variable” positions may represent sequencing error hotspots or unannotated paralogous sequences and were filtered out. We considered that remaining nucleotide positions sequenced at high coverage (>150 total reads) and with a RAF between 1–10% and 90–99% in Mauritania-I represented positions where a previously unreported minor clone differed from the major clone. We then reconstructed the haploid genome sequence of this second (minor) clone using the minor allele at these positions (i.e., we assumed that only one minor strain was present in this sample).

In addition to the Mauritania-I strain data generated by the Broad Institute [3], we analyzed blood samples from four additional monkey blood samples collected during the generation of the Mauritania-I strain (derived from the initial patient infection) and Mauritania-II strain (derived from a relapse of the same patient) [26]. We also analyzed blood directly collected from the patient during the relapse. The quality of the DNAs (frozen since 1995 for most samples) and the lack of leukocyte depletion before freezing prevented whole genome sequencing. We therefore designed primers to amplify 38 SNVs distributed across the *P*. *vivax* genome and for which we observed two alleles in the Mauritania-I Broad Institute sequence data (S2 Table). Each primer was designed to include a 5’ oligonucleotide tail for barcoding and high-throughput sequencing (see below). We amplified each locus with the following conditions: 94°C for 3 min; 40 cycles of 94°C for 45 sec, 56°C for 45 sec and 72°C for 45 sec; and final extension at 72°C for 3 min. We pooled the 38 amplification products obtained from each blood sample together, purified the DNA pools with Qiagen QIAquick columns and labeled them with an individual oligonucleotide barcode (i.e., one barcode per blood sample) by a second amplification (with the same conditions as previously but with only 10 cycles) using primers targeting the 5’ oligonucleotide tail and containing the Illumina adapter sequence and the unique barcode sequence. The barcoded samples were then pooled together at equal DNA concentrations and sequenced simultaneously on an Illumina MiSeq to generate 32,283,840 paired-end reads of 150 bp (4.4–10.8 million pairs per sample). We mapped the reads on the Salvador I reference genome sequence using bowtie2 and analyzed allelic variations at the SNVs targeted. We discarded from our analysis 11 out of the 38 targeted SNVs due to allelic dropout or insufficient read coverage (<100 X).

## Results

### Host switch was not associated with systematic changes in the *P*. *vivax* genome sequence

**Fig 1:**
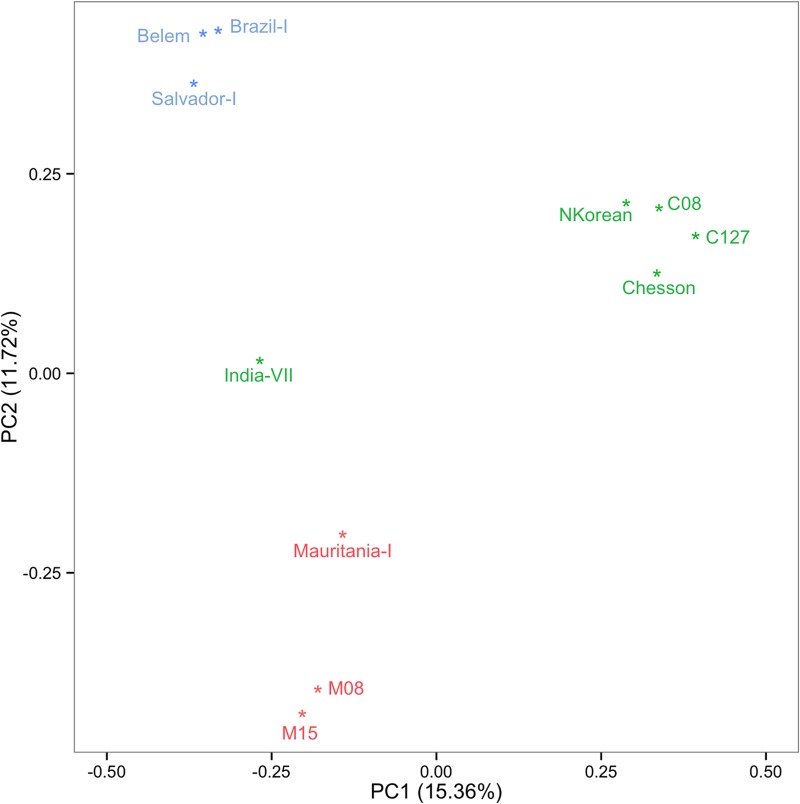
Genomic relationships among *P*. *vivax* isolates. Principal component analysis based on 81,328 SNVs in four field isolates (C127, C08, M08 and M15) and seven monkey-adapted strains (Salvador-I, Brazil-I, Belem, Chesson, North Korea, India-VII and Mauritania-I) for which an entire haploid genome sequence could be reconstructed. The sample names are colored by their geographic origin: blue for Central and South America, green for Asia and red for Africa.

We analyzed 19.7 million nucleotide positions (87% of the Salvador I reference genome sequence) that have been previously sequenced at more than 20 X in seven monkey-adapted strains and six field isolates and identified 140,949 variable positions. We refer to these variable nucleotide positions as single nucleotide variants (SNVs) as they may include variants that occurred during the adaptation and propagation of the strains in New World monkeys as well as single nucleotide polymorphisms (SNPs). In all monkey-adapted strains and four of the field isolates, one clone of *P*. *vivax* accounted for >80% of all *P*. *vivax* sequences enabling reconstruction of the entire haploid genome sequence for this clone. To assess whether adaptation to a new host induced systematic genomic changes, we first performed a principal component analysis of all dominant clones using all SNVs identified. Interestingly, *P*. *vivax* parasites clustered according to their geographic origin and not to the host species from which the sample was obtained (Fig. 1). Further analyses of the first ten principal components (accounting for 94% of the variance) did not reveal any clustering of samples according to their host. This observation indicated that, at the genome level, the host switch was not a major determinant of the genetic diversity.

Even if host switch did not alter the genetic diversity of *P*. *vivax* at the genome-scale, it is possible that a few critical protein coding genes or regulatory elements were systematically modified during the parasite passage from human to monkey hosts. We therefore examined every SNV throughout the genome and tested whether its alleles segregated according to the host. Throughout 19.7 Mb covered by more than 20 high quality reads in all samples, we did not find a single variant (out of 140,949 SNVs) for which one allele was fixed in all monkey-adapted strains and the other allele was fixed in all human field isolates (e.g., a position where all monkey-adapted strains would carry an A and all human isolates a T). Analyses of DNA sequence insertions, deletions or inversions [7][8] also failed to reveal any DNA sequence rearrangement systematically present in all samples from one group and absent from all samples from the other. Overall, our analyses suggested that adaptation to a New World monkey host did not induce systematic genomic changes nor did it leave any consistent signature in the *P*. *vivax* genome among the strains evaluated here.

### Long-term propagation of *P*. *vivax* in monkeys did not lead to mutation accumulation

Once adapted to a different host, *P*. *vivax* strains can be propagated for years through successive infections of New World monkeys. We therefore wanted to determine whether this propagation could lead to genetic changes. If monkey-adapted strains accumulate mutations during propagation, we would expect that they differed more from each other or from a set reference than field isolates. In contrary, our results showed that there were, on average, 36,297 nucleotide differences (16,683–47,597) between a given monkey-adapted strain and the Salvador-I reference genome sequence and 40,730 differences (38,520–45,306) between human isolates and the reference (p = 0.4).

Another way to test whether long-term propagation in New World monkeys result in the accumulation of mutations is to compare genome sequences from the same strain generated from DNA isolated years apart. We have independently [5] produced sequencing data from the Salvador-I strain used for generating the reference genome sequence [25]. Out of the ~12.2 million bases covered by 20 reads or more in our data and after filtering out ~360 kb of repetitive or potentially paralogous regions (see [5] for details), we observed 3,116 possible SNVs (i.e., positions where >10% of the reads differed from the reference allele) between the genomes of this same strain collected at two time points. However, there were only 8 positions where >90% of the reads generated differed from the reference Salvador I sequence (note that these figures are slightly different from those presented in [5] as we used here a better read mapping algorithm). It is important to note that these differences represented a combination of sequencing errors and possible genuine differences. Overall, these observations suggested that propagation in New World monkeys was unlikely to lead to accumulation of many mutations in the *P*. *vivax* genome.

### Sub-telomeric deletion of chromosome 7

**Fig 2:**
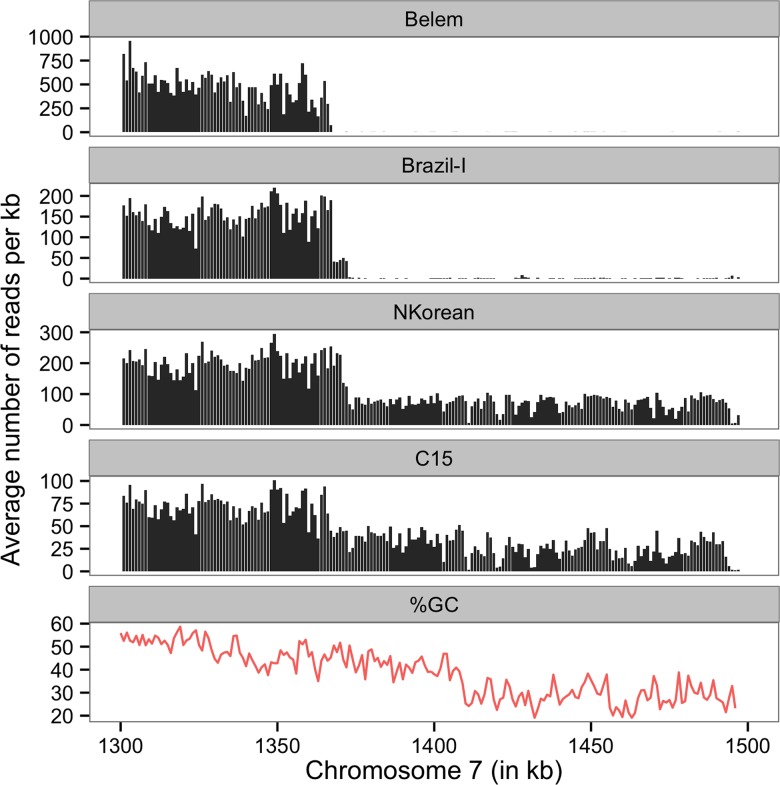
Shared telomeric deletion. The figure shows a ~120 kb deletion indicated by the decrease in sequence coverage (y-axis, in reads per bp) at the telomeric end of chromosome 7 (x-axis in 1,000 bp). The sequence coverage is displayed, from top to bottom, for three monkey-adapted strains (Belem, Brazil-I and North Korea) and one Cambodian field isolate (C15). The bottom track shows the variation in GC content along this region. The lower coverage in North Korea and C15 indicates that only some of the parasites carry the deletion. Note also that the deletion boundary is different in different samples.

**Fig 3:**
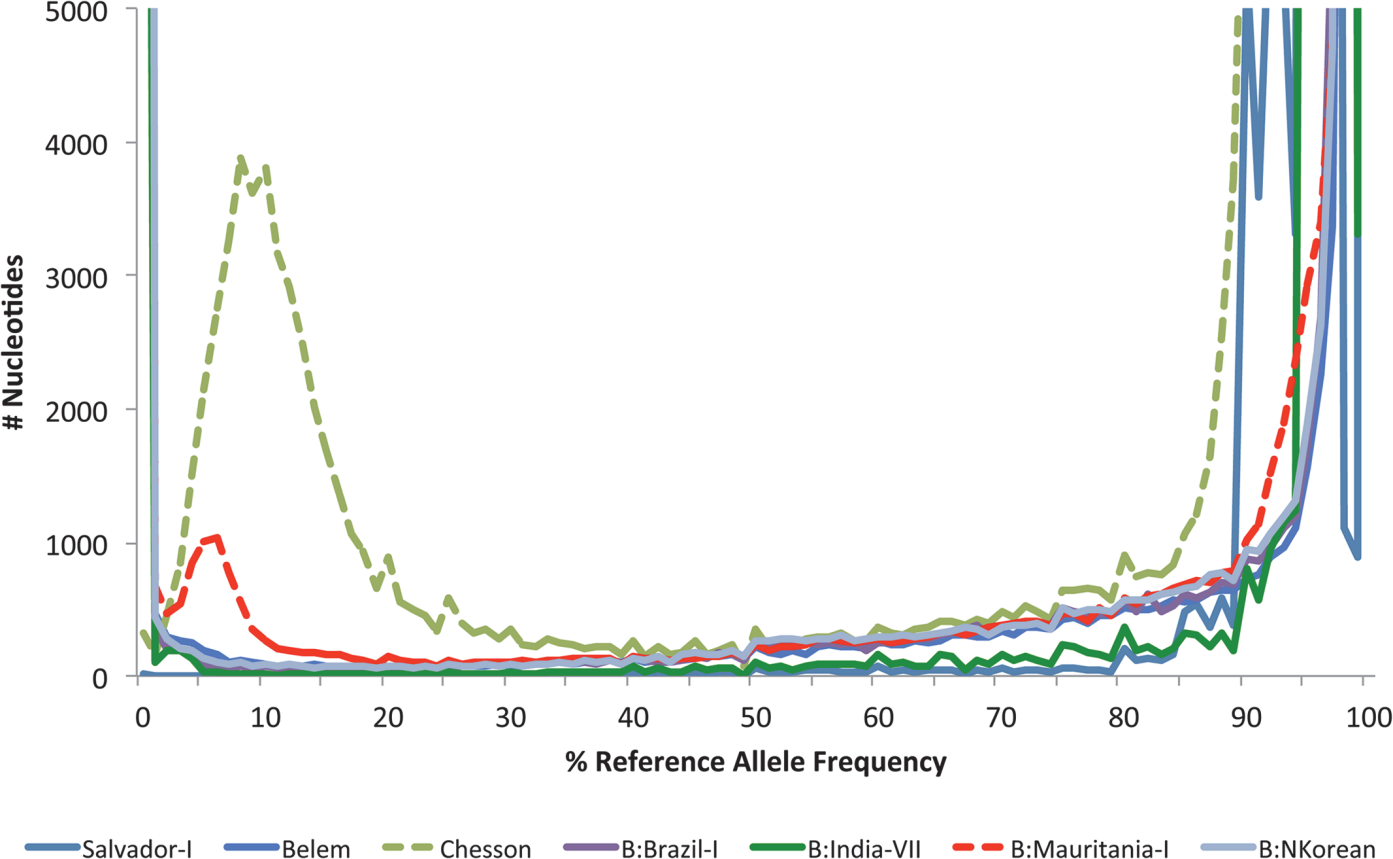
Distribution of the Reference Allele Frequency (RAF) in monkey-adapted strains sequenced to date. The graph shows the number of variable positions (y-axis) in a given sample according to the proportion of reads carrying the reference (i.e., Salvador I) allele (x-axis, in %). For most monkey-adapted strains the distribution is U shaped consistent with the present of a single haploid clone. However, the RAF distributions for Mauritania-I (in dotted red) and Chesson (in dotted green) indicate the presence of a second clone. The RAF for the human isolates mentioned in the manuscript is presented in S1 Fig.

In three of the seven monkey-adapted strains (Belem, Brazil-I and North Korean), we noticed that very few (if any) reads mapped to a 130 kb region at the subtelomeric end of chromosome 7 (Fig. 2). While telomeric and subtelomeric regions are enriched in repeated sequences and therefore difficult to assemble, resequence and analyze, this particular deletion extended far beyond the typical repeat- and AT-rich region and was successfully sequenced in other *P*. *vivax* strains. In addition, the GC content along this subtelomeric region gradually decreases with the most abrupt change (from ~40% to ~28% GC) occurring around position 1,411,000, roughly 35 kb downstream of the deletion boundary (Fig. 2). The deleted region contains 22 annotated protein coding genes including a cytoadherence linked asexual protein (CLAG, PVX_086930), an early transcribed membrane protein (ETRAMP, PVX_086915), a Phist protein (PVX_086910), ten hypothetical proteins and nine *vir* genes. In the Belem and Brazil-I strains, no sequence reads could be aligned to this region suggesting that the entire end of the chromosome had been deleted. The exact demarcation of the deletion did not appear to be identical between these samples, with the deletion starting at base ~1,367,000 in the Belem strain and 6 kb later, at base ~1,373,000, in the Brazil-I and North Korean strains (Fig. 2). This could indicate independent deletion events or continuous trimming of the telomere. Evidence of this subtelomeric deletion in the North Korean strain was supported by a significant, but not complete, reduction in coverage (~75% less reads), suggesting that, within the North Korean strain, some parasites carried the deletion while some had the entire subtelomeric sequence. Interestingly, the reference allele frequency (RAF) profile for the North Korean strain (Fig. 3, light blue) suggested that the two clones in this sample (with or without the deletion) were otherwise genetically identical. This observation suggested that the subtelomeric deletion occurred recently in a clonal population of parasites and that the North Korean strain of *P*. *vivax* is not genetically homogeneous anymore. It is important to note that this telomere shortening was not exclusive to monkey-adapted strains but was also observed in one of the minor clones of a field isolate from Cambodia (C15, Fig. 2).

### Monkey-adapted strains can consist of a population of genetically different parasites

In a previous study we showed that the Salvador-I and Belem displayed reference allele frequency (RAF) distributions consistent with the presence of a single clone [5]. For these samples, all reads covering a given genome position either carried a nucleotide identical to the reference allele or all carried a same but different nucleotide (the alternative allele), with minor alleles represented by less than 5% of the reads likely representing sequencing errors. This pattern was also observed in three out of four monkey-adapted strains sequenced by the Broad Institute [3] (Fig. 3).

In contrast, in the Chesson sample, we detected the presence of a second clone that accounted for approximately 10% of all reads (Fig. 3). At all positions that harbored two alleles for this sample, the minor allele was always identical to the Salvador-I reference allele. In addition, we did not observe a single position with a RAF of 0% (which occurs when both clones are identical and differ from the reference genome) suggesting that, throughout the entire genome, the minor clone sequence never differed from the Salvador-I reference genome sequence. These observations suggested that the Chesson sample we sequenced had likely been contaminated by Salvador-I DNA.

**Fig 4:**
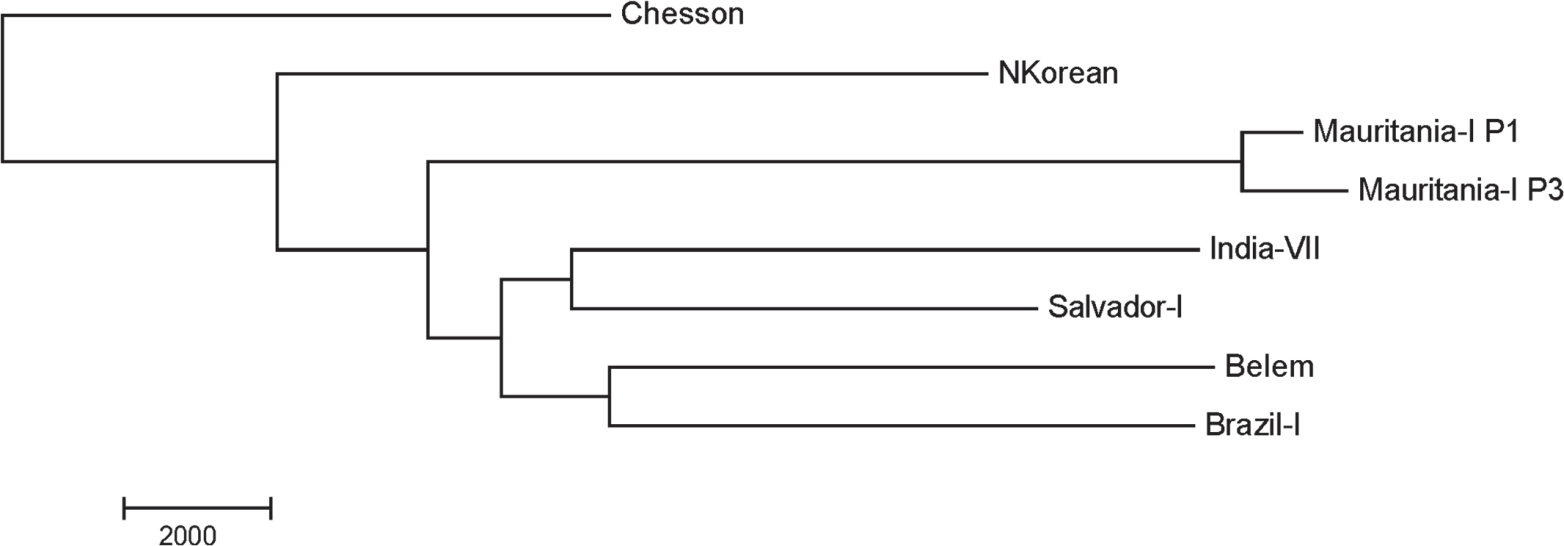
Relationships among different monkey-adapted strains. The figure shows a neighbor-joining tree based on the number of nucleotide differences between samples using all variable positions throughout the genome. Note that the DNA sequences of the two clones identified in the Mauritania strain are much more closely related (i.e., shorter branch lengths) than any other two sequences.

**Fig 5:**
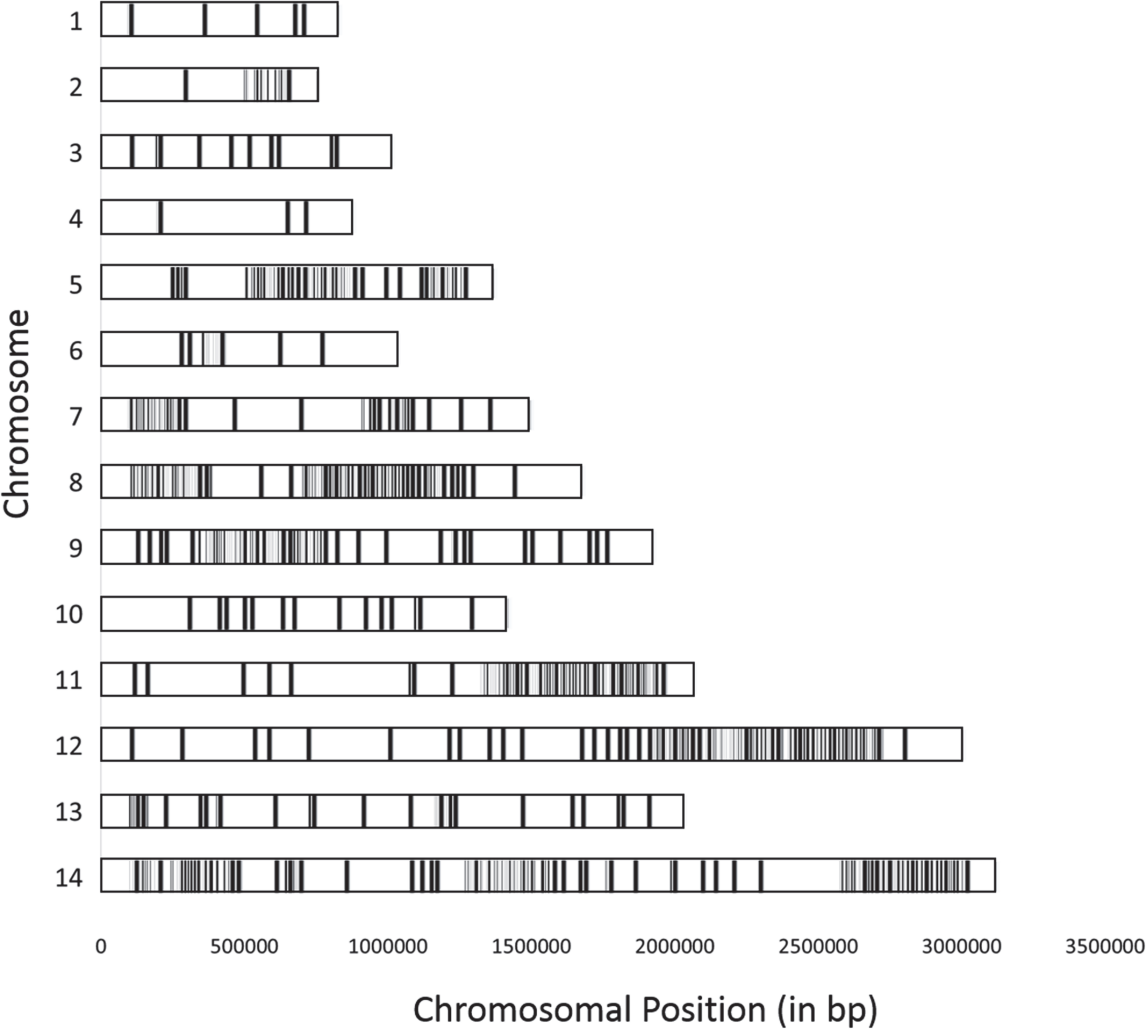
Genomic distribution of the nucleotide differences between the clones present in the Mauritania-I sample. Each grey bar represents one single nucleotide difference between the two clones detected in the Mauritania-I genome sequence data and is displayed according to its position (x-axis, in bp) along one of the *P*. *vivax* chromosome (from chromosome 1 on top to chromosome 14 at the bottom). Note that 1,969 out of the 2,255 nucleotides differences (87%) between the two clones were clustered in 153 regions accounting for 3.78 Mb (or 20% of the genome).

**Fig 6:**
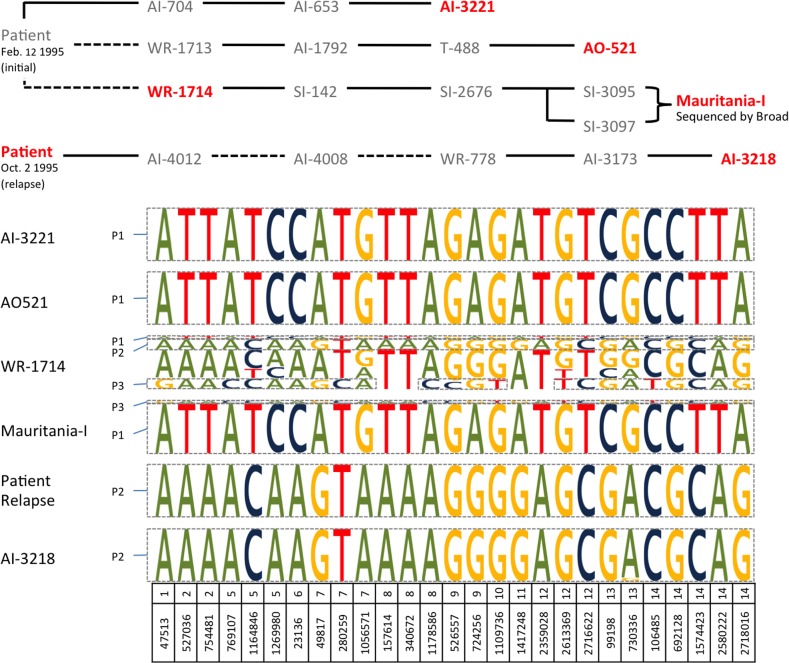
Complexity of infection in the Mauritania-I and Mauritania-II samples. The top panel shows the passage history of the Mauritania-I (starting with the initial patient infection of February 12, 1995) and Mauritania-II (starting with the October 2 relapse) strains (adapted from Collins et al., 1998). The six samples analyzed in this study are indicated in red. Solid black lines represent infections propagated in either *Aotus nancymaae* (AI, AO and WR) or *Saimiri boliviensis boliviensis* (SI) monkeys through injections of infected erythrocytes. Dashed lines represent passage through mosquitoes and propagation by sporozoites. The lower panel shows genotypes of the different clones present in each of the Mauritania samples analyzed (all samples are monkey-adapted strains but the “Patient Relapse” which is a clinical sample). The height of each allele represents its relative frequency in each sample and the alleles are organized based on the haplotypes inferred for each clone. Note that the allele frequencies in WR-1714 likely indicate the presence of one or more supplementary clones in addition to P1, P2 and P3.

The RAF spectrum of the Mauritania-I strain (Fig. 3) also clearly indicated the presence of a minor clone accounting for ~5% of the *P*. *vivax* sequences. Overall, we identified 2,255 nucleotide positions where the two clones present in the Mauritania-I sample differed. This number of differences was much lower than we would expect for two unrelated clones (typically around 30,000 nucleotide differences) and suggested that these clones were likely related (Fig. 4). Analysis of the spatial distribution of these genetic differences revealed that the SNVs differentiating the two clones of Mauritania-I were not randomly spread throughout the genome (as would be expected from a unrelated clone) but instead appeared to be clustered in distinct “blocks” (Fig. 5): 1,969 out of 2,255 differences (87%) were located in 153 regions ranging from 5 kb to 165 kb (and accounting for 3.78 Mb or 20% of the genome sequence). One possible explanation for this block pattern is that several *P*. *vivax* clones were present in the original patient infection and that they recombined during the passage through *Anopheles* mosquitoes in the laboratory (Fig. 6) and that the Mauritania-I sample originally sequenced by the Broad Institute is a mixture of a parental and a recombinant clone.

To confirm the presence of multiple clones in the Mauritania-I sample, we analyzed blood samples collected at different time points along the generation of the Mauritania-I strain (see Fig. 6 and Methods for details). We selected 38 SNVs differentiating the major and minor clones present in the Mauritania-I genome sequence and located in the recombinant blocks. We genotyped these SNVs (see Methods) in three monkey *P*. *vivax* infection samples derived from the initial malarial episode (AI-3221, AO-521 and WR-1714, which was infected by sporozoites from mosquitoes fed on the patient’s blood) and two samples from a subsequent relapse of the same patient (blood from the relapsing patient and, after serial passage in New World monkeys, from a later monkey-adapted stabilate, AI-3218). These samples are collectively referred to as Mauritania-I (for the samples derived from the initial infection) and Mauritania-II (for the samples derived from the relapse).

At each of the 27 position successfully genotyped, the monkey samples AI-3221 and AO-521 showed 100% of the reads carrying the same allele indicating that these samples were infected by a single clone (referred to as P1). The sample from the patient relapse and the monkey sample derived from this relapse (AI-3218) also showed genotypes consistent with infection by parasites with the same single genotype as one another. However, this genotype was different from the P1 genotype noted in AI-3221 and AO-521 at 19 of the 27 successfully genotyped SNVs (Fig. 6) indicating that the patient’s relapse parasites and the parasites passaged through AI-3218 were a distinct clone (referred to as P2). Finally, the genotypes generated from the sample WR-1714 showed two alleles at 24 out of 27 positions indicating the presence of multiple *P*. *vivax* clones in this sample. This observation confirmed that the two clones detected in the Mauritania-I genome sequence data were genuine (and not the result of a laboratory contamination) since the passage lineage of the sample sequenced by the Broad Institute derives from WR-1714 (Fig. 6). WR-1714 displayed genotypes consistent with the presence of both P1 and P2 clones as well as a third clone (P3) at a much lower frequency (<5%). Overall, our analyses are consistent with the presence of at least three clones (P1, P2, and P3) in the original infection, a single clone (P2) in the patient’s relapse blood specimen (and the subsequent infected monkeys) and the presence of two clones (a predominant P1 clone and a minor recombinant clone of P1 and P3) in the sample sequenced by the Broad Institute (Fig. 6). Note that, since we selected SNPs differentiating the recombinant clone from the major clone (P1) from the Mauritania-I genome data, the recombinant genotype is identical to its parental genotype (P3) at these markers.

## Discussion

The main purpose of this study was to determine whether the adaptation of the human malaria parasites *P*. *vivax* to New World monkey hosts resulted in systematic genetic or genomic changes. Overall, our analyses suggested that monkey-adapted strain genomes were not significantly altered and remained representative of the original *P*. *vivax* parasite genomes circulating in the blood of the infected patient. In particular, we did not detect any fixed nucleotide differences between field isolates and monkey-adapted strains suggesting that the host switch did not lead to systematic genetic changes. Our analyses relied on the comparison of existing monkey-adapted *P*. *vivax* genomes to those of field isolates. A more elegant and straight-forward approach would be to directly compare the genomes of the same *P*. *vivax* strain generated from DNA isolated from the original patient and from an infected New World monkey after adaptation. Unfortunately, few laboratories are able to perform such host switch and they do so irregularly, and no matched DNA pairs from previous adaptations were available for genome sequencing.

We have also tested whether monkey-adapted strains accumulate mutations during continuous propagation in monkeys. The mutation rate during asexual reproduction of *P*. *vivax* remains unknown and long-term culture studies similar to those performed in *P*. *falciparum* [29] are not necessarily comparable to *in vivo* propagation. However, analysis of the genome of the monkey-adapted Salvador-I strain sequenced from two New World monkeys separated by at least five consecutive passages revealed a small number of putative genetic changes suggesting a low asexual mutation rate (note that these differences could also originate from sequencing errors). Importantly, most of these nucleotide differences between the Salvador-I reference genome and our later sequence were only supported by a small proportion of the reads and only 8 nucleotide differences were supported by 90% or more of the reads (out of 12 Mb sequenced at more than 20 X in Salvador I). This observation suggested that, despite likely population bottlenecks occurring during the propagation of the Salvador-I strain in different monkeys, few novel mutations (if any) have drifted to fixation and that most of the possible differences observed are only present in a subset of the otherwise clonal parasite population. Studies including multiple passages will be required to confirm these findings and provide a rigorous estimate of the mutation rate during asexual reproduction.

One limitation of our analyses is that we excluded regions of the *P*. *vivax* genome where high DNA sequence homology or unannotated paralogous sequences greatly complicates unambiguous read mapping and SNP calling. While we analyzed here 87% of the *P*. *vivax* reference genome, it is possible that unidentified mutations occurred, during adaptation and propagation of these strains in monkeys, in the remaining non-unique regions of the *P*. *vivax* genome. Similarly, we did not consider short indels for technical reasons and these might represent another source of possible genetic differences unaccounted for in our study.

During our analyses, we observed a large deletion at the subtelomeric end of chromosome 7 in three out of the seven monkey strains, as well as in one Cambodian field isolate. While telomeres are typically difficult to sequence and assemble (and are partially missing in the Salvador-I reference genome sequence), this deletion mostly included unique DNA sequences and contained little repeated sequences. Similar subtelomeric deletions have been reported in *P*. *falciparum*, both in field isolates and *in vitro* cultures (e.g., [22,23]). Interestingly, the chromosome 7 subtelomeric deletion displayed different boundaries in different samples suggesting that i) it resulted from independent events that occurred in the *P*. *vivax* population prior to adaptation to New World monkeys or ii) that the telomere was slowly being eroded. In addition, in the North Korean strain we observed genetic heterogeneity for this rearrangement suggesting that a proportion of the parasites in the sequenced sample carried the deletion while the rest of them had the full-length chromosomal sequence. This observation suggested that the subtelomeric loss was recent in this strain (i.e., post adaptation to monkeys) and that it remained polymorphic in this otherwise clonal parasite population. This finding also raised questions regarding the presumed genetic homogeneity of monkey-adapted strains.

One technical factor may artificially influence the heterogeneity of the strains: DNA samples collected from multiple individual monkeys infected with the same strain are often pooled together to obtain enough genetic material for genome sequencing. This procedure may result in laboratory contamination with another strain, especially since these strains are not differentiable without the use of genetic markers. For example, we detected a contamination of the Chesson sample by the Salvador-I strain. Such cross-contamination could have important consequences: sequencing a particular gene may, for example, reveal two different DNA sequences and suggests that there are multiple copies of that gene in this strain.

Finally, we observed in the Mauritania-I sample sequenced by the Broad Institute [3] evidence of genetic heterogeneity, with the presence of at least two genetically distinct clones. Analysis of additional Mauritania-I samples confirmed that multiple clones were present in the original patient infection and revealed that different clones became isolated (or dominant) in different monkeys during the propagation. This observation raises important concerns on the use of monkey-adapted *P*. *vivax* strains as different aliquots of the same monkey-adapted strain might actually contain genetically different parasites and therefore might respond differently in *in vitro* or *in vivo* assays (e.g., of drug resistance, infection efficiency or virulence). On the other hand, our study illustrates the potential advantages of applying genomic tools to studies of monkey-adapted strains. Identification of multiple clones in a sample is traditionally conducted by genotyping a small number of microsatellites (typically between 5 and 10), which does not have the sensitivity necessary to differentiate closely related clones or identify clones making up less than 10% of the parasites [30]. The resources provided by genomic data now enable genotyping of several dozen of highly informative SNPs and might help in solving phenotypic discrepancies among samples from the same monkey-adapted strain. In addition, the observation of a recombinant clone in the Mauritania-I sample sequenced by the Broad Institute illustrates how application of genomic tools could guide the generation of *P*. *vivax* genetic crosses which could lead to major advances in gene mapping in *P*. *vivax* (but see also [31]).

The development and maintenance of monkey-adapted *P*. *vivax* strains has and will continue to be an essential tool for the study of this important malaria parasite. While we have highlighted some of the hidden problems of monkey-adapted strains, our study also provides great prospects for studying this important resource. The extensive information generated by genome sequencing provides numerous genetic markers that can easily be genotyped in a given sample to monitor the identity, complexity and purity of a given strain and improve studies of monkey-adapted strains.

## Supporting Information

S1 Table Summary of the samples included in the study. The table shows the origin, source of DNA, and sequencing statistics (including accession numbers) for all samples. Note that all parasites originally infected humans and that “Host” only refers to the host the parasite DNA was obtained from.
(DOCX)

S2 Table SNV genotyping in Mauritania strain. The table indicates the position of the targeted SNV, the primers used and the number of reads obtained for each amplicon and each sample.
(DOCX)

S1 Fig Distribution of the Reference Allele Frequency (RAF) in human isolates. The graph shows the number of variable positions (y-axis) in a given sample according to the proportion of reads carrying the reference (i.e., Salvador I) allele (x-axis, in %).
(TIF)
