# Supplementary material for: Comparative Analysis of Field-Isolate and Monkey-Adapted Plasmodium vivax Genomes
Source: PLoS Negl Trop Dis. 2015 Mar 13;9(3):e0003566. doi: 10.1371/journal.pntd.0003566 (PMC4358935; doi:10.1371/journal.pntd.0003566)
Supplement: S1 Table — The table shows the origin, source of DNA, and sequencing statistics (including accession numbers) for all samples. Note that all parasites originally infected humans and that “Host” only refers to the host the parasite DNA was obtained from. (DOCX) [file pntd.0003566.s001.docx]

Supplemental Table S1

| **Isolate name** | **Host** | **Country** | **SRA** | **Type** | **# Reads generated** | **# Reads aligned** | **Sequencing design** | **Average Coverage** | **Reference** | **Note** |
| --- | --- | --- | --- | --- | --- | --- | --- | --- | --- | --- |
| M08 | human | Madagascar | SRX187092 | WGS | 215,643,747 | 32,737,022 | 100 bp paired end | 235 | Chan et al., 2012 |  |
| M15 | human | Madagascar | SRX266275 | WGS | 76,891,834 | 11,154,838 | 100 bp paired end | 82 | Menard et al., 2013 |  |
| M19 | human | Madagascar | SRX187093 | WGS | 85,703,544 | 17,788,686 | 100 bp paired end | 127 | Chan et al., 2012 |  |
| C08 | human | Cambodia | SRX187072 | WGS | 231,291,984 | 14,773,862 | 100 bp paired end | 110 | Chan et al., 2012 |  |
| C15 | human | Cambodia | SRX187091 | WGS | 79,414,201 | 11,022,204 | 100 bp paired end | 78 | Chan et al., 2012 |  |
| C127 | human | Cambodia | SRX187089 | WGS | 211,061,945 | 61,052,160 | 100 bp paired end | 437 | Chan et al., 2012 |  |
| Salvador-I | monkey | Brazil | SRX188940 | WGS | 215,743,944 | 2,926,207 | 100 bp paired end | 21 | Chan et al., 2012 |  |
| Belem | monkey | Peru | SRX188939 | WGS | 81,446,663 | 61,411,645 | 100 bp paired end | 454 | Chan et al., 2012 |  |
| Chesson | monkey | Papua New Guinea | SRX266375 | WGS | 171,808,907 | 13,025,883 | 100 bp paired end | 98 | Hester et al., 2013 |  |
| Brazil-I | monkey | Brazil | SRR332569 | WGS | 133,804,387 | 31,107,871 | 101 bp paired end | 197 | Neafsey et al., 2012 |  |
|  |  |  | SRX092662 | WGS | 46,377,215 | 9,897,987 | 101 bp paired end | 46 | Neafsey et al., 2012 | only used for assessing errors |
|  |  |  | SRR340133 | WGS | 34,036,283 | 7,320,036 | 76 bp paired end | 41 | Neafsey et al., 2012 | only used for assessing errors |
| India-VII | monkey | India | SRR332913 | WGS | 95,900,222 | 9,934,008 | 101 bp paired end | 60 | Neafsey et al., 2012 |  |
| Mauritania-I | monkey | Mauritania | SRR332413 | WGS | 130,982,283 | 53,681,876 | 101 bp paired end | 340 | Neafsey et al., 2012 |  |
|  |  |  | SRX092459 | WGS | 36,123,329 | 13,919,900 | 101 bp paired end | 65 | Neafsey et al., 2012 | only used for assessing errors |
|  |  |  | SRR340129 | WGS | 45,606,818 | 14,877,884 | 76 bp paired end | 73 | Neafsey et al., 2012 | only used for assessing errors |
| North Korea | monkey | North Korea | SRR332562 | WGS | 132,927,529 | 38,295,377 | 101 bp paired end | 245 | Neafsey et al., 2012 |  |
|  |  |  | SRX092667 | WGS | 52,601,299 | 14,201,750 | 101 bp paired end | 66 | Neafsey et al., 2012 | only used for assessing errors |
|  |  |  | SRR340091 | WGS | 46,472,094 | 10,405,179 | 76 bp paired end | 48 | Neafsey et al., 2012 | only used for assessing errors |
|  |  |  |  |  |  |  |  |  |  |  |
| AI-3221 | monkey | Mauritania | SRX685966 | Locus Seq | 1,366,193 | 1,222,904 | 150 bp paired end | 5,940 | this study |  |
| AO-521 | monkey | Mauritania | SRX687257 | Locus Seq | 1,187,553 | 1,047,092 | 150 bp paired end | 6,127 | this study |  |
| WR-1714 | monkey | Mauritania | SRX687261 | Locus Seq | 2,852,157 | 2,620,869 | 150 bp paired end | 6,286 | this study |  |
| AI-3218 | monkey | Mauritania | SRX687262 | Locus Seq | 1,788,655 | 1,673,576 | 150 bp paired end | 6,248 | this study |  |
| Relapse | human | Mauritania | SRX687263 | Locus Seq | 1,424,639 | 1,295,959 | 150 bp paired end | 5,394 | this study |  |
