# Supplementary material for: Comparative Analysis of Field-Isolate and Monkey-Adapted Plasmodium vivax Genomes
Source: PLoS Negl Trop Dis. 2015 Mar 13;9(3):e0003566. doi: 10.1371/journal.pntd.0003566 (PMC4358935; doi:10.1371/journal.pntd.0003566)
Supplement: S2 Table — The table indicates the position of the targeted SNV, the primers used and the number of reads obtained for each amplicon and each sample. (DOCX) [file pntd.0003566.s002.docx]

Supplemental Table S2

| **Informative** | **Chr** | **Position** | **Forward** | **Reverse** | **Length** | **5' Start** | **3' End** | **AI-3218** | **AI-3221** | **AO-521** | **Relapse** | **WR-1714** |
| --- | --- | --- | --- | --- | --- | --- | --- | --- | --- | --- | --- | --- |
| X | 1 | 47513 | CCAATACAGCCAAAGCCAA | TTCAATGTTTTGAGCAGGTTG | 121 | 47424 | 47545 | 41488 | 53235 | 37058 | 34778 | 48407 |
|  | 1 | 361432 | GGAGCAAAGTCCGCAAAA | GTATCACGTGCGCGCTTT | 123 | 361351 | 361474 | 69976 | 49037 | 44608 | 47033 | 122455 |
|  | 2 | 293524 | GCTCCTCTTTCAGTTGGCA | GGACCACATGCACCACG | 128 | 293427 | 293555 | 78489 | 67548 | 55762 | 9567 | 147809 |
| X | 2 | 527036 | AAAATTATGGGTCGCGCA | TTGGACACTGCTCTCGTTCT | 123 | 526972 | 527095 | 77566 | 67410 | 57497 | 29156 | 126119 |
| X | 2 | 754481 | AGGACGAAGTTGTAGGTTTCG | GGTGTCGCACATTATTCCG | 107 | 754421 | 754528 | 87637 | 81840 | 62217 | 50127 | 135351 |
|  | 3 | 107993 | CCCACAAAGTTATGCGAGC | GCGTCAACACGGGTTTGT | 119 | 107941 | 108060 | 82662 | 69415 | 53917 | 27884 | 141830 |
| X | 5 | 769107 | TGGGAAATCAATGCTGTGC | CTCAACACACTTGGGGGC | 105 | 769053 | 769158 | 35999 | 39298 | 23486 | 35470 | 53002 |
| X | 5 | 1164846 | CCATTGGGTGGTTGATGG | ATCGAACGGAACAGCCAC | 109 | 1164806 | 1164915 | 72370 | 51475 | 41530 | 68767 | 144263 |
| X | 5 | 1269980 | CCAATTTTCCCACTGTTCATC | GGGGAGATTGGGTAAAATCC | 125 | 1269929 | 1270054 | 84460 | 57115 | 57937 | 148719 | 102331 |
| X | 6 | 23136 | ATTATTGCAGCCCCCACA | CGCCTTCTACACTGCCTG | 112 | 23059 | 23171 | 1321 | 56143 | 60454 | 188 | 1550 |
|  | 6 | 371442 | CCTAGTTGAACGCGTGGC | TCGTGGAGCTGCAAAAGA | 126 | 371370 | 371496 | 83327 | 55489 | 56205 | 163533 | 55600 |
| X | 7 | 49817 | TAAAATTGCGCCCAAGGA | CCACGTTTCCCGTCTGAA | 124 | 49781 | 49905 | 33407 | 10629 | 10648 | 2464 | 17089 |
| X | 7 | 280259 | GGGCAATGCTCAGTGGAC | GTTTGGGACCCCATCCTC | 113 | 280226 | 280339 | 67782 | 52864 | 58269 | 143534 | 132157 |
|  | 7 | 697371 | AAAACCACACGCGGAGAA | GGGAGGTGGCAAGGTTGT | 127 | 697274 | 697401 | 79450 | 57579 | 52434 | 162752 | 157019 |
| X | 7 | 1056571 | CCTTTGGACCCACCACAG | CAACGGACGACCTGTTCC | 105 | 1056524 | 1056629 | 69093 | 40441 | 35530 | 72185 | 63249 |
| X | 8 | 157614 | GACACAGCGGAAGCCCTA | TGTTGCGGACTCCCTCTT | 129 | 157547 | 157676 | 76872 | 50424 | 52025 | 74637 | 110661 |
| X | 8 | 340672 | AGGAGGGGGATTCAAAGG | CCCCCTCTCCCTTTTAGC | 97 | 340615 | 340712 | 45119 | 33242 | 22866 | 261 | 47857 |
| X | 8 | 1178586 | ACGCAAAAGCGCTCGTAT | CGCGAATGCATACGTAAAA | 125 | 1178507 | 1178632 | 73415 | 64537 | 38643 | 38235 | 84532 |
| X | 9 | 526557 | GCCCACGCAGGTACACTC | TCGGCGTTTTGTCTCTCC | 115 | 526504 | 526619 | 65269 | 53110 | 46278 | 20943 | 113398 |
| X | 9 | 724256 | CCGTGTTCCGTTCAGCTT | TGCTACACCCTTCTGCCC | 100 | 724224 | 724324 | 64555 | 44594 | 46912 | 81446 | 146368 |
|  | 9 | 1286388 | GTTTCCGCCCCTTTGACT | AATAGCAGGAACGGCGTG | 111 | 1286336 | 1286447 | 79109 | 48711 | 50074 | 44150 | 129658 |
|  | 10 | 922798 | GAAAGCGCCATTTGAAGC | GCTTGAGCCTGTTTGCGT | 125 | 922742 | 922867 | 74086 | 51037 | 43065 | 40877 | 134825 |
| X | 10 | 1109736 | CCCCCTGACGTAAAAGAACA | TGTCACGCCCATCAGTCT | 130 | 1109667 | 1109797 | 73976 | 53410 | 39611 | 14489 | 71600 |
|  | 11 | 162574 | GCATATACGTTCGCATAAACG | TGCGTATTTTTGCAAAGGC | 125 | 162506 | 162631 | 81026 | 51796 | 52574 | 69303 | 129391 |
|  | 11 | 584756 | GCAAGTGCATGGGCGTAT | TGACGGGTTAGCGGGTTA | 103 | 584708 | 584811 | 88659 | 65237 | 63295 | 167661 | 207363 |
| X | 11 | 1417248 | TGGATTAGCTGCAACGGG | CGCACATAGGAGGGAGGA | 117 | 1417190 | 1417307 | 90757 | 61263 | 60188 | 59644 | 179235 |
|  | 12 | 1401871 | CATGCGTGTGAATGCTGC | GTGCCACGTGTGGGTACA | 130 | 1401792 | 1401922 | 2794 | 3471 | 2537 | 2221 | 1879 |
| X | 12 | 2359028 | CCCTCGTGTTTGTCACTGG | AACACCTTTGCTAACCGCC | 115 | 2358954 | 2359069 | 62708 | 56519 | 53204 | 56138 | 141468 |
| X | 12 | 2613369 | CGATTGCAGCTACACGGA | CGCATAGTCTAAGCCCACG | 121 | 2613331 | 2613452 | 64340 | 53771 | 54664 | 74056 | 128432 |
| X | 12 | 2716622 | CAGCCAAAATGGCGAACT | GGACAACTTCCACGTCGC | 126 | 2716554 | 2716680 | 76054 | 55772 | 53795 | 54129 | 116176 |
| X | 13 | 99198 | TTTTTCATTTCGTTACCCCC | TGCATGCAGGAAGGTGAA | 125 | 99126 | 99251 | 95513 | 69030 | 60157 | 25837 | 168941 |
|  | 13 | 366254 | ACTTGGCTGTTGGCAAGC | AGCAGATGCGGAAGATGC | 106 | 366197 | 366303 | 57780 | 36153 | 27232 | 85473 | 66856 |
| X | 13 | 730336 | GCGAAATGATGAAGAGCGA | CCCCAGGTTCATGCTGAC | 101 | 730305 | 730406 | 77965 | 58200 | 62519 | 44446 | 134384 |
| X | 14 | 106485 | ACGAAAAACAAAGAGAAAGCC | TTGTTTGAAATGGCTGTTTG | 130 | 106445 | 106575 | 73492 | 51439 | 43835 | 50187 | 90820 |
| X | 14 | 692128 | ATAAACCGCACCACCACG | CACGTCAAACACGTAGGGC | 126 | 692049 | 692175 | 58640 | 37389 | 32020 | 69168 | 141859 |
| X | 14 | 1574423 | TCAAATTTCGGACCAGCC | GGGGCTCCAAATCAAAGG | 117 | 1574384 | 1574501 | 78383 | 53151 | 52034 | 146949 | 149341 |
| X | 14 | 2580222 | GGCCAGGCATGGCTACTA | GCCACTTTCCAACTCGTGA | 124 | 2580134 | 2580258 | 107689 | 75162 | 49964 | 251 | 125470 |
| X | 14 | 2718016 | ACAGGAGCATCGTCCGAG | CCGCAGGAAGGGAAGAAG | 104 | 2717973 | 2718077 | 102085 | 74433 | 56368 | 80662 | 151694 |
