# Supplementary figures and images for: Comparative Analysis of Field-Isolate and Monkey-Adapted Plasmodium vivax Genomes
Source: PLoS Negl Trop Dis. 2015 Mar 13;9(3):e0003566. doi: 10.1371/journal.pntd.0003566 (PMC4358935; doi:10.1371/journal.pntd.0003566)

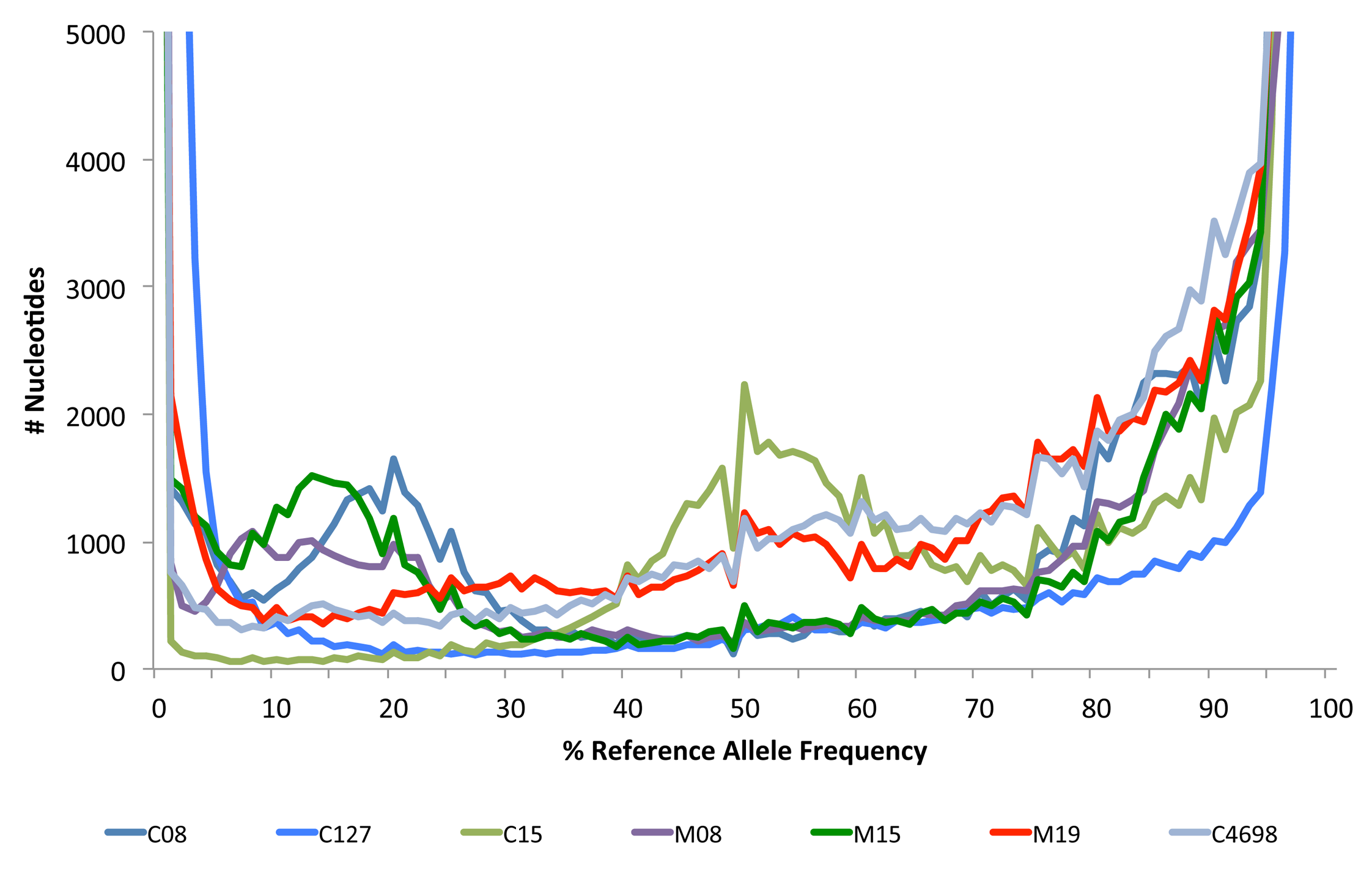

Supplement: S1 Fig — The graph shows the number of variable positions (y-axis) in a given sample according to the proportion of reads carrying the reference (i.e., Salvador I) allele (x-axis, in %). (TIF) [file pntd.0003566.s003.tif]
